# Rotated spiral RARE for high spatial and temporal resolution volumetric arterial spin labeling acquisition

**DOI:** 10.1016/j.neuroimage.2020.117371

**Published:** 2020-09-12

**Authors:** Fanny Munsch, Manuel Taso, Li Zhao, R. Marc Lebel, Arnaud Guidon, John A. Detre, David C. Alsop

**Affiliations:** a Division of MRI Research, Department of Radiology, Beth Israel Deaconess Medical Center, Harvard Medical School, 330 Brookline Avenue, Boston, MA 02215, USA; b Diagnostic Imaging and Radiology, Children’s National Hospital, Washington, DC, USA; c Global MR Applications and Workflow, GE Healthcare, Calgary, AB, Canada; d Global MR Applications and Workflow, GE Healthcare, Boston, MA, USA; e Departments of Neurology and Radiology, University of Pennsylvania, Philadelphia, PA, USA

**Keywords:** MRI, Arterial spin labeling, Perfusion, Resting-state networks, Brain connectivity, Compressed sensing

## Abstract

**Background:**

Arterial Spin Labeling (ASL) MRI can provide quantitative images that are sensitive to both time averaged blood flow and its temporal fluctuations. 3D image acquisitions for ASL are desirable because they are more readily compatible with background suppression to reduce noise, can reduce signal loss and distortion, and provide uniform flow sensitivity across the brain. However, single-shot 3D acquisition for maximal temporal resolution typically involves degradation of image quality through blurring or noise amplification by parallel imaging. Here, we report a new approach to accelerate a common stack of spirals 3D image acquisition by pseudo golden-angle rotation and compressed sensing reconstruction without any degradation of time averaged blood flow images.

**Methods:**

28 healthy volunteers were imaged at 3T with background-suppressed unbalanced pseudo-continuous ASL combined with a pseudo golden-angle Stack-of-Spirals 3D RARE readout. A fully-sampled perfusion-weighted volume was reconstructed by 3D non-uniform Fast Fourier Transform (nuFFT) followed by sum-of-squares combination of the 32 individual channels. Coil sensitivities were estimated followed by reconstruction of the 39 single-shot volumes using an L_1_-wavelet Compressed-Sensing reconstruction. Finally, brain connectivity analyses were performed in regions where BOLD signal suffers from low signal-to-noise ratio and susceptibility artifacts.

**Results:**

Image quality, assessed with a non-reference 3D blurring metric, of full time averaged blood flow was comparable to a conventional interleaved acquisition. The temporal resolution provided by the acceleration enabled identification and quantification of resting-state networks even in inferior regions such as the amygdala and inferior frontal lobes, where susceptibility artifacts can degrade conventional resting-state fMRI acquisitions.

**Conclusion:**

This approach can provide measures of blood flow modulations and resting-state networks for free within any research or clinical protocol employing ASL for resting blood flow.

## 1. Introduction

Arterial Spin Labeling (ASL) perfusion MRI is widely used to provide a quantitative, time-averaged measure of blood flow and brain function for research and clinical applications. A typical acquisition will involve several minutes of data collection to reduce thermal and physiologic noise by averaging and, for some methods, to acquire sufficient spatial encoding to reconstruct an image (Alsop et al., 2015). Such time averaging potentially discards a wealth of information on vascular regulation and brain function that could be inferred from temporal blood-flow fluctuations. These fluctuations could be induced by blood pressure or blood gas challenges (Zhao et al., 2009) or brain stimulation paradigms (Aguirre et al., 2002), or simply observed through correlations between brain voxels (Dai et al., 2016, Zhao et al., 2019) or with other physiologic measures at rest (Yan et al., 2016, O’Gorman et al., 2013, Tong et al., 2013). These additional data could be acquired through single-shot acquisitions, but often at the cost of spatial resolution, image quality, or physiologic noise suppression.

Single-shot acquisition for ASL can be achieved either by rapid 2D multi-slice approaches or by accelerated 3D methods. Though 2D acquisitions have been possible for many years and have been used for most of the important work on functional ASL (Edelman et al., 1994, Kwong et al., 1995, Alsop and Detre, 1996), they do have limitations. Even with simultaneous multi-slice excitation (Kim et al., 2013, Feinberg et al., 2013), acquiring sufficient 2D slices to image the entire brain causes a spread in labeling delay and T_1_ decay that compromises sensitivity and introduce sensitivity biases. Additionally, reduction of physiologic and motion related noise by background suppression is more complicated and less effective than in 3D methods (Shao et al., 2018, Vidorreta et al., 2013). For these reasons, 3D acquisitions have been recommended in a recent consensus statement (Alsop et al., 2015). However, single-shot remains a challenge for 3D acquisitions. Techniques such as stack of spiral (SOS) Rapid Acquisition with Relaxation Enhancement (Hennig et al., 1986) (RARE, otherwise known as Fast Spin Echo or Turbo Spin Echo) (Dai et al., 2008, Vidorreta et al., 2014, Ye et al., 2000) or gradient and spin echo (GRASE) (Günther et al., 2005) can be accelerated by parallel imaging (Chang et al., 2017), but high levels of acceleration typically introduce artifacts and amplify noise. High image quality with parallel acceleration by introduction of spatial and temporal regularization terms has recently been reported for GRASE ASL acquisition (Spann et al., 2019), but the effect of temporal regularization on sensitivity to fluctuations was not evaluated. Further improvement in parallel imaging acceleration has been shown when k-space is undersampled such that aliasing artifacts are spatially incoherent and reconstruction is performed with regularization that prioritizes sparsity of a transformed version of the image, a method known as Compressed Sensing (CS) (Lustig et al., 2007, Otazo et al., 2010, Zhao et al., 2015). However, maintaining spatially incoherent sampling in gradient accelerated 3D acquisitions can be challenging.

Golden-angle rotation (Winkelmann et al., 2007) across slices is an appealing approach to spatially incoherent 3D k-space sampling that is applicable to fast gradient methods like spiral. It has been used successfully for temporal and spatial acceleration in cardiac applications (Feng et al., 2014), primarily using radial trajectories and gradient echo acquisition. This approach has also been applied to ASL angiography (Zhou et al., 2017). Golden-angle rotation of spirals has been employed in 3D (Lim et al., 2019, Deng et al., 2016, Turley and Pipe, 2013) and 4D (Bastkowski et al., 2018) gradient echo acquisitions and in multi-shot SOS RARE ASL without acceleration (Li et al., 2016), but not to our knowledge in single-shot accelerated SOS RARE. Here, we implement and evaluate a rotated SOS acquisition combined with CS reconstruction for temporally resolved 3D ASL and apply the method to imaging of resting-state fluctuations.

## 2. Methods

### 2.1. Theory

**Fig. 1.**
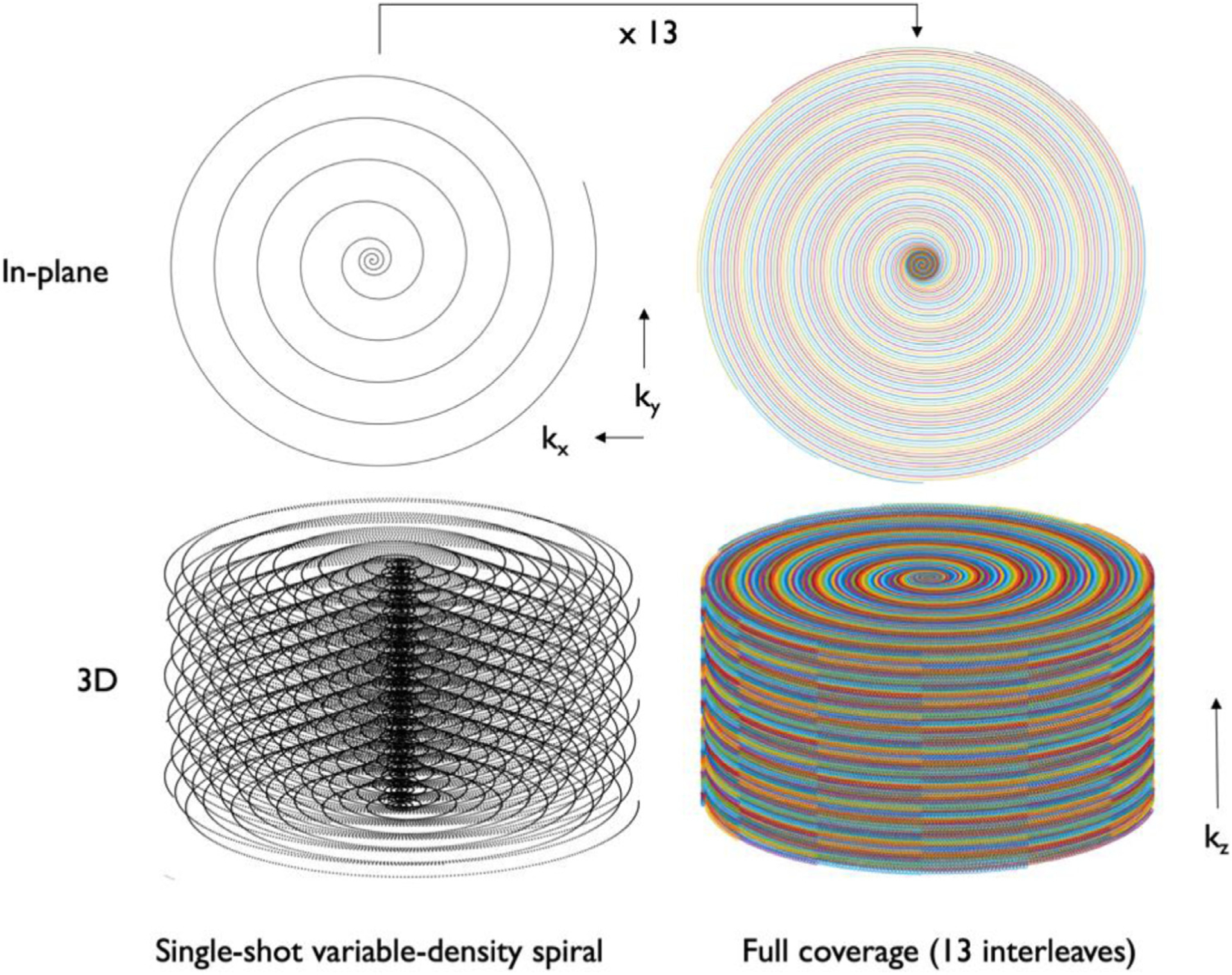
Illustration of the k-space sampling using interleaved spirals with pseudo golden-angle rotations showing a single-shot spiral and the whole acquisition in-plane (top) and 3D coverage (bottom).

Reconstruction of single-shot volumes from unrotated interleaved acquisitions by parallel imaging and CS is not optimal, however (Zhou et al., 2017). To improve reconstruction of single-shot spirals while maintaining full k-space sampling across interleaves, a different sampling strategy was adopted based on golden-angle rotations. A variable-density spiral waveform was designed to provide full k-space center sampling (inner 5% of radius) while providing an 8 fold undersampling of the outer k-space using an optimization method accounting for the gradient constraints (King et al., 1995). This individual spiral waveform was then rotated across slice encodes and excitations by 10 *π*/13 = 138.46° (an approximation (Lim et al., 2019) of the golden angle, 137.51°), i.e. $\theta =\frac{10\pi }{13}\left(\text{slice}\;\text{encode}+\text{interleave}-1\right)$ with slice encodes from -number of slices/2 to number of slices/2 −1 and interleaves from 1 to the number of interleaves. This rotation retains the sampling benefits of the golden angle, but because it repeats every 13 rotations, it produces an uniform sampling across the slice direction after 13 excitations that can be reconstructed by more available slice Fourier Transform and 2D nonuniform FFT (Fig. 1). The cyclic rotation also reduces memory demands if rotated gradient waveforms are stored in memory.

Our modified encoding strategy promises significant benefits as it spreads undersampling gaps within the spiral and slice direction helping simultaneously in collecting a high-quality fully-sampled image by standard regridding and incoherent sampling for parallel-imaging informed CS reconstruction of single-shot volumes. Depending on the temporal resolution requirements, it also provides flexibility by potentially reconstructing volumes with either high temporal / low spatial sampling or the reverse, while simultaneously providing an average high spatial resolution perfusion volume.

### 2.2. Experiments

Studies were performed in *N* = 28 volunteers (17 Females, ages ranging from 19 to 39) recruited according to a protocol approved by the institutional committee on clinical investigations; all subjects provided written informed consent. We report results from a baseline scan, part of a larger protocol testing sensitivity to drug modulations of brain activity. Drug effects will be reported elsewhere.

All scanning was performed on a 3T scanner (Discovery MR750, GE Healthcare, Waukesha, WI), using body coil RF transmission and a 32-channel head coil for reception (Nova Medical, Wilmington, MA). Prior to ASL acquisition, fast anatomic localizer images were acquired followed by 1 × 1 × 1mm resolution spoiled gradient-echo T_1_-weighted (GE BRAVO) 3D images.

#### 2.2.1. ASL preparation

**Fig. 2.**
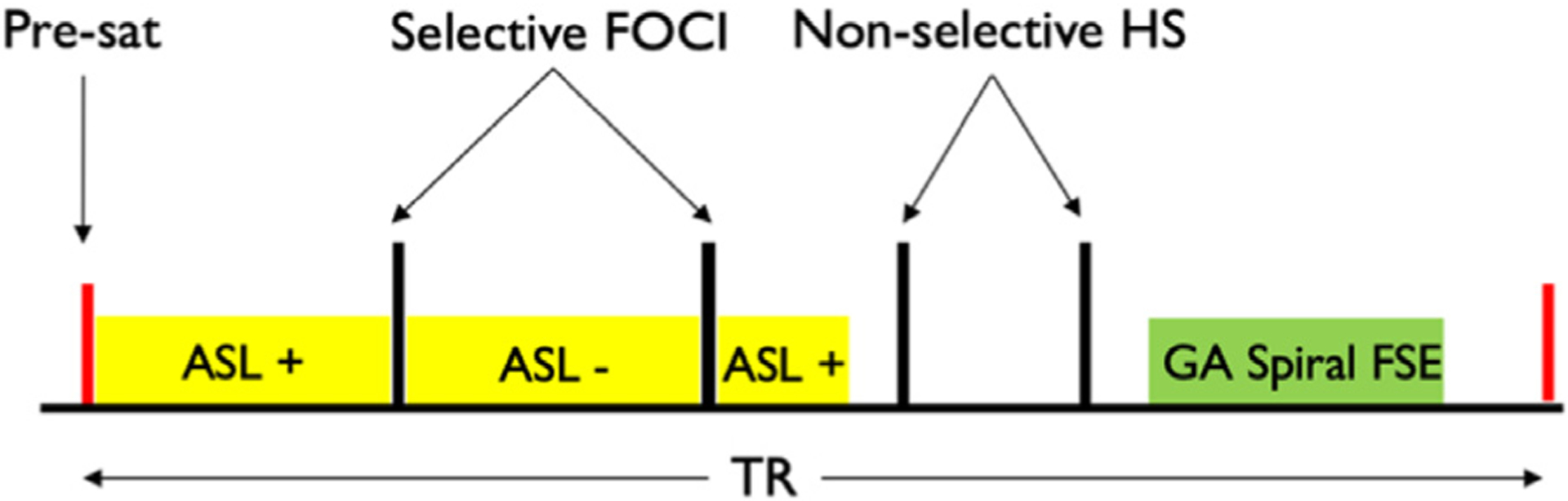
A schematic of the pseudo-continuous ASL preparation with interleaved background suppression used in this study. Label (−) and control (+) pulsing are switched after each spatially selective FOCI background suppression pulse. Approximate golden-angle spiral fast spin echo readout (GA Spiral FSE).

Golden-angle (GA) rotated and conventional acquisitions were performed with identical preparation. A background-suppressed unbalanced pCASL scheme with interleaved labeling and background suppression for flexible prescription of labeling duration / post-labeling delay (PLD) was employed (Dai et al., 2012) as described in Fig. 2. The labeling was applied with an average RF amplitude of B_1_ = 1.8 *μ*T for 1.8s followed by a 1.8s PLD using average and peak gradients of 0.7 / 4.5mT/m to improve robustness towards off-resonance and pulsatility effects (Zhao et al., 2017). Background suppression was achieved by repeated spatially selective presaturation ending at 3.6s before imaging, spatially selective FOCI inversion pulses applied at 2848 and 1546ms before imaging, and then two non-selective hyperbolic secant pulses at 605 and 131ms. Inferior saturation was applied at 299ms before imaging to null late inflowing blood. Labeling was switched between label and control after each selective inversion. These timings were derived by an optimization procedure to achieve background suppression to less than 1% of the unrelaxed magnetization (Maleki et al., 2012). For quantification, an additional M_0_ reference volume was acquired without background suppression but with spatial presaturation 2s before imaging.

#### 2.2.2. ASL acquisitions

Two separate acquisitions were performed: a conventional interleaved SOS and the new GA rotated acquisition proposed above. Both employed 32, 4mm slice centric encoded echoes using a reduced refocusing flip angle train optimized for constant echo amplitude (Alsop, 1997), neglecting T_2_ decay, of 111.1°, 72.2°, 62.8°, 60.9° and then approximately constant at 62°.

The GA rotated sequence employed a variable density spiral of 6ms duration sampled at 1536 points with a receiver bandwidth of 125 kHz. The maximum k-space radius reached corresponds to an in-plane 2.88mm resolution. The echo spacing and echo time were 12.9ms and the sequence was repeated with a TR of 6.6s. As described above, gradient waveforms were rotated by an approximate golden angle for each slice encode step and for each interleaved excitation. Label and control pairs with identical spiral rotation were acquired in successive excitations and repeated for a total of 39 interleaved pairs with spiral rotation. Since the spiral waveforms repeat every 13 rotations, this can be viewed as 3 averages of 13 interleaves. A 13 interleave M_0_ reference image with presaturation at 2s before imaging was also acquired at the end of the sequence. The total acquisition time was approximately 9 min.

The conventional acquisition employed a stack of 8 interleaves (Alsop et al., 2015, Dai et al., 2012, Dai et al., 2013, Zhao et al., 2017) (also recommended in the ADNI protocol http://adni.loni.usc.edu/adni-3/), a 4ms gradient waveform reaching a maximum k-space radius corresponding to 3.46mm sampled at 1024 points with a receiver bandwidth of 125 kHz, a TE of 10.9ms and a TR of 6s. 3 averages and a M_0_ reference image were acquired in 5 min.

In the manuscript we will employ the following naming for our two main acquisitions:

- **conventional** for no variable density with conventional rotation across interleaves and no rotation across slice encodes acquisition
- **Golden-angle (GA) rotated** for variable density with full golden-angle rotation (rotation across interleaves and slices encodes) acquisition, which refers to our sequence.

### 2.3. Image reconstruction

Data from the ASL acquisitions were saved as raw echo signals for offline reconstruction. The reconstruction pipeline was implemented using MATLAB (R2017b, MathWorks, Natick, MA) and the Berkeley Advanced Reconstruction Toolbox (BART) (Uecker et al., 2015)). All fully sampled acquisitions, including the average GA rotated perfusion-weighted subtraction, the conventional acquisition perfusion-weighted subtraction, and the M_0_ reference images, were reconstructed in a 128 × 128 × 32 matrix (FOV, 24 × 24cm^2^; voxel size, 1.9 × 1.9 × 4mm^3^) by performing a 3D non-uniform Fast Fourier Transform (nuFFT) with L_2_ regularization (*λ*=0.01) followed by sum-of-squares combination of the 32 individual channels. This reconstruction was preferred to the more standard slice Fourier Transform followed by 2D nuFFT or regridding because it followed the same reconstruction path and geometry as the CS single-shot reconstruction.

**Fig. 3.**
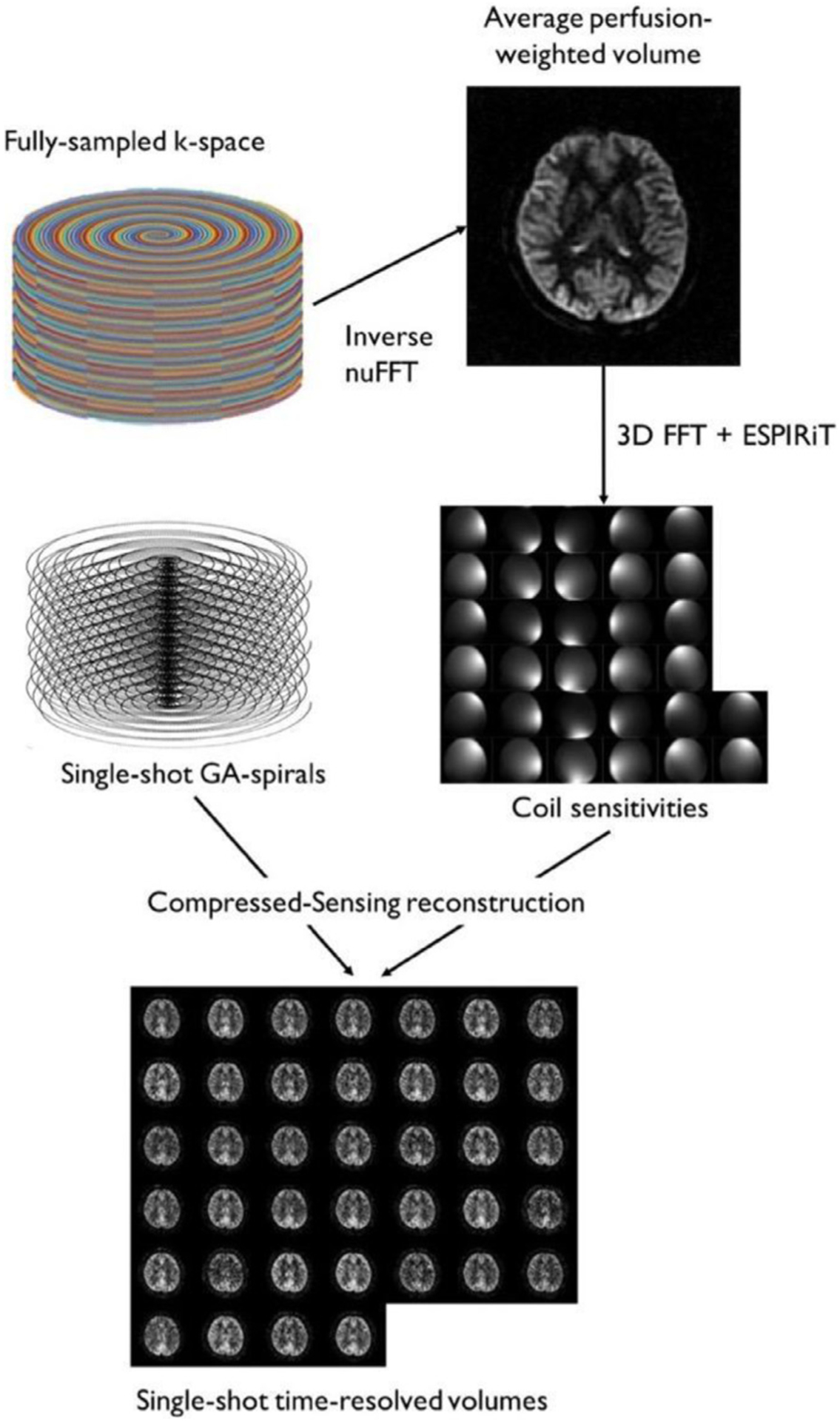
The image reconstruction pipeline for single-shot accelerated imaging. Conventional nuFFT reconstruction of all k-space data over time produced a high-quality time averaged ASL image that could be used to determine coil sensitivities. These sensitivities were then used to reconstruct the single-shot acquisitions using an iterative CS algorithm.

The single-shot compressed sensing reconstruction pipeline is displayed in Fig. 3. A complex subtraction in k-space was performed between the control (M_control_) and label (M_label_) data to form a perfusion-weighted k-space (*d*M = M_control_ – M_label_). To reduce slice direction blurring (Zhao et al., 2018), raw data were corrected for signal decay between echoes by multiplying each echo with a correction factor derived from simulations of the reduced flip angle RARE sequence and assuming T_2_=100ms and T_1_=1400ms. A spherical Fermi type filter (Zhao et al., 2018) was also applied to control Gibbs ringing and reduce noise contribution from the corners of k-space. To estimate individual coil sensitivities required for the parallel imaging informed CS reconstruction, a 3D FFT was performed on the 128 × 128 × 32 time averaged ASL volume from the rotated spiral data, followed by ESPIRiT (Uecker et al., 2014) coil-sensitivity calibration (eigenvalue threshold = 0.8, null-space threshold = 0.01). We chose to use the perfusion-weighted subtractions to estimate coil sensitivities because the M_0_ reference scan is not systematically acquired in clinical routine if CBF quantification is not required.

The 39 single-shot ASL volumes (13 interleaves × 3 averages = 39) were then reconstructed using an L_1_-regularized parallel-imaging CS reconstruction with wavelet as sparsifying transform and data scaling turned off to enable absolute quantification. *λ*_1_, the regularization factor for L_1_, was set to 2000 (in arbitrary units). This value was selected empirically, based on multiple *λ* reconstructions in several subjects, as a balance between noise reduction and image quality. The iterative minimization problem was solved using the Alternative Direction Methods of Multipliers (ADMM) algorithm in 100 iterations.

To assess the impact of interleaving approach on single-shot image quality, we also reconstructed images from a single interleave of the more standard spiral trajectory without rotation across slices. Because the saved data for this acquisition was already summed across the 3 averages, reconstruction of the 3-shot average data for each single interleave was performed. Single interleave data from the GA rotated acquisition were also reconstructed after threefold averaging. Reconstruction of both data sets was performed with the identical CS algorithm and parameters described for the single-shot acquisitions.

### 2.4. Analyses

Image quality of the accelerated acquisitions was assessed by comparing the images resulting from the conventional acquisition, the full time-averaged images produced by reconstruction of the GA rotated acquisition using all interleaves and averages, and the time average of the single-shot reconstructed GA rotated images. ASL perfusion-weighted images were compared, rather than quantitative perfusion images, because image division and thresholding required by the quantification may degrade the image quality. To assess the image quality of the average GA rotated perfusion-weighted (PW) volumes, we compared it to the conventional PW volume by computing difference maps, expressed in percentage of the whole brain mean signal. We also calculated the normalized root-mean-square errors (NRMSE), defined as the RMSE divided by the RMS signal within the brain, and a metric of 3-dimensional blurring (Crété-Roffet et al., 2007) for both acquisitions across all volunteers. Quantification for both acquisitions was performed by calculating cerebral blood flow (CBF) maps using the two-compartment model (Parkes, 2005) and mean whole-brain CBF was compared. Paired t-tests were performed, using R (version 3.5), to assess the significance of the differences between both acquisitions.

Similarly, difference image, NRMSE and 3D blurring were computed between the average GA rotated perfusion image and the temporal average of the 39 single-shot images in order to estimate the degradation of image quality induced by the parallel-imaging informed CS reconstruction. Paired t-tests were performed to assess the significance of the differences.

Finally, in order to assess the benefit of rotating spiral between interleaves, we reordered the data acquired with the GA rotated acquisition for one subject in order to simulate a variable density with partial golden-angle rotation (no rotation between slice encodes but rotation across interleaves) acquisition, referred to as **unrotated** in the following.

### 2.5. Extraction of resting-state networks

As the temporal resolution of the GA rotated acquisition was 13.2s (2*TR) and resting-state fluctuations are known for being low-frequency fluctuations (<0.25 Hz), the temporal resolution of this acquisition is sufficient to detect resting-state networks.

The time series of single-shot ASL volumes (*N*=28 subjects) were pre-processed using SPM12 (Statistical Parametric Mapping, Wellcome Trust Center for Neuroimaging, London, UK) and custom MATLAB scripts, as follows. First, we segmented the 3D T_1_-weighted volumes to obtain a gray matter (GM) map used later for registration and normalized them to the MNI152 space using non-linear registration. We motion corrected the single-shot ASL volumes using rigid registration to the mean. Then, we registered those volumes to the native GM map using rigid registration. We normalized them to the MNI152 space by applying the deformation field calculated for the normalization of the 3D T_1_-weighted volumes. We smoothed the volumes first slightly with a 2D Fermi-filter in k-space and then more substantially with a 4mm FWHM Gaussian kernel and removed the first volume of each time-series because it can be affected by transients. The binary brain mask, *mask_ICV*, from SPM12 was applied to the smoothed volumes. A global spatial mean signal was calculated for each volume and a scale factor was applied to achieve equal time averaged global signal across volumes and subjects. These images were then analyzed for connectivity with global normalization across time within subjects.

Resting-state correlation analyses were performed using the CONN toolbox (Whitfield-Gabrieli and Nieto-Castanon, 2012). We set up the analysis using the default parameters but turned off some that are applicable to BOLD signal but not ASL: CSF, WM and effect of rest as confounds in denoising and scan weighting by the hemodynamic response function. We first performed ICA analysis using the default parameters (fastICA for individual-level analysis and GICA3 for group-level analysis) to assess the capability of this new sequence to extract resting-state networks. We investigated 11 independent components (ICs) after exploring a number of ICS between 10 and 15 and compromising between separation of ICs and significant ICs. Type I error was controlled through the use of voxel-level and cluster-level thresholds (respectively, *p* < 0.01 FDR and *p* < 0.05 cluster-size familywise error corrected). Then, we performed seed-to-voxel connectivity analyses using *a priori* regions of interest (ROIs) where resting-state (rs) BOLD analyses can be challenging due to low signal to noise ratio (SNR) and susceptibility artifacts. Thus, we chose to study the medial prefrontal cortex (MPFC), part of the default-mode network (DMN), and the left amygdala for which rs-BOLD signal is impacted by susceptibility artifacts. Studying networks involving deep nuclei, like the amygdala, is also of interest because BOLD signal is reduced in them due to low blood volume. Cluster-level T-contrasts were obtained using non-parametric statistics (permutation/randomization tests). Type I error was controlled through the use of voxel-level and cluster-level thresholds (respectively, *p* < 0.001 uncorrected and *p* < 0.01 cluster-size familywise error corrected).

### 2.6. Data and code availability statements

Anonymized imaging data will be shared upon request from any interested and qualified investigator after completing a Data Sharing Agreement with Beth Israel Deaconess Medical Center.

## 3. Results

### 3.1. Image quality of rotated spiral images reconstructed from full data

**Fig. 4.**
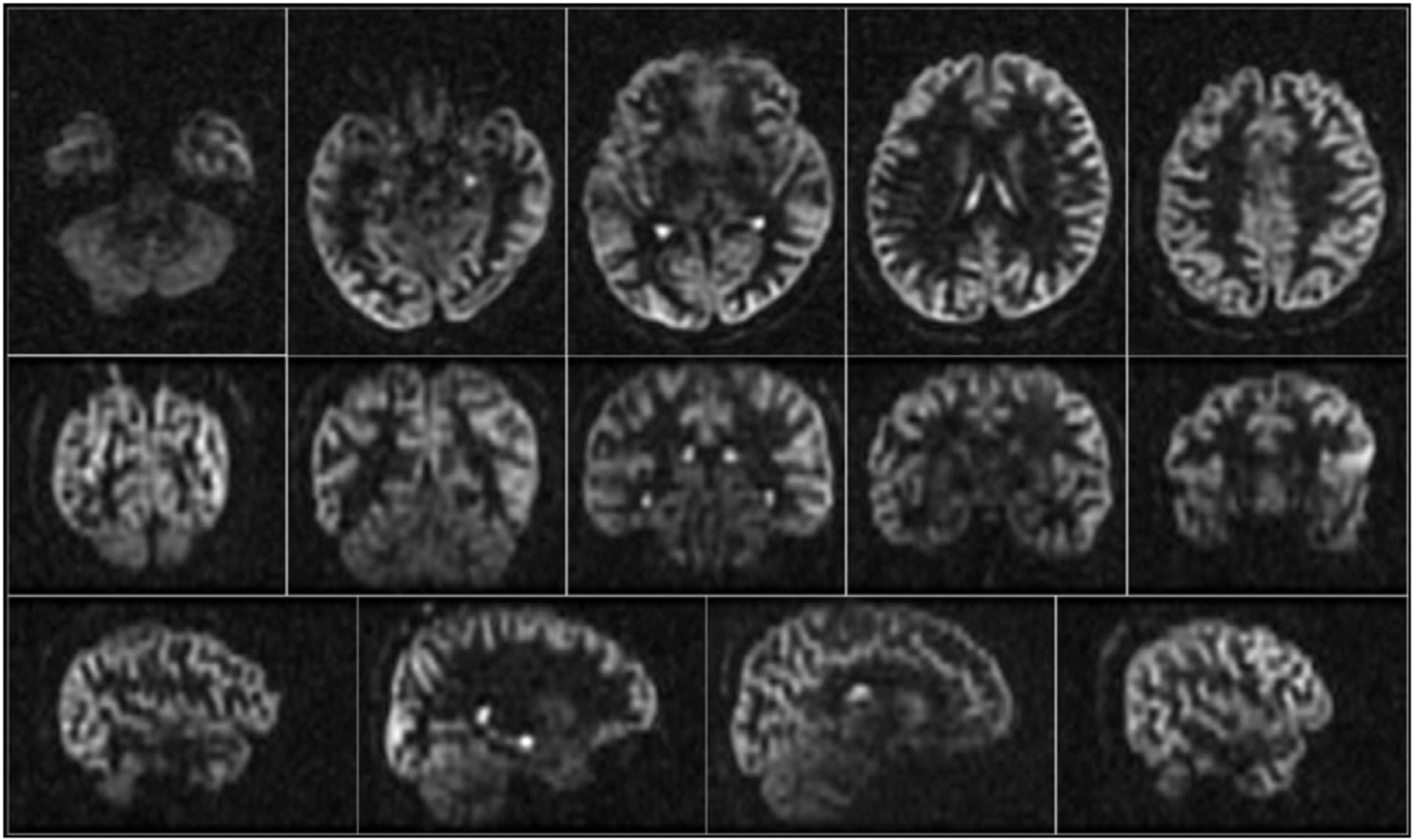
Example time averaged ASL difference images acquired with the GA rotated acquisition from a representative subject.

Full time-averaged perfusion images reconstructed from all the interleaves of the GA rotated acquisition displayed excellent image quality and were free from artifacts (Fig. 4). In particular, there was no indication that rotation of spirals within each excitation affected refocusing of the ASL signal across echoes, as might occur in the presence of uncompensated eddy currents or other imperfections. Slice direction T_2_ blurring appears largely corrected by the echo scaling prior to reconstruction.

**Fig. 5.**
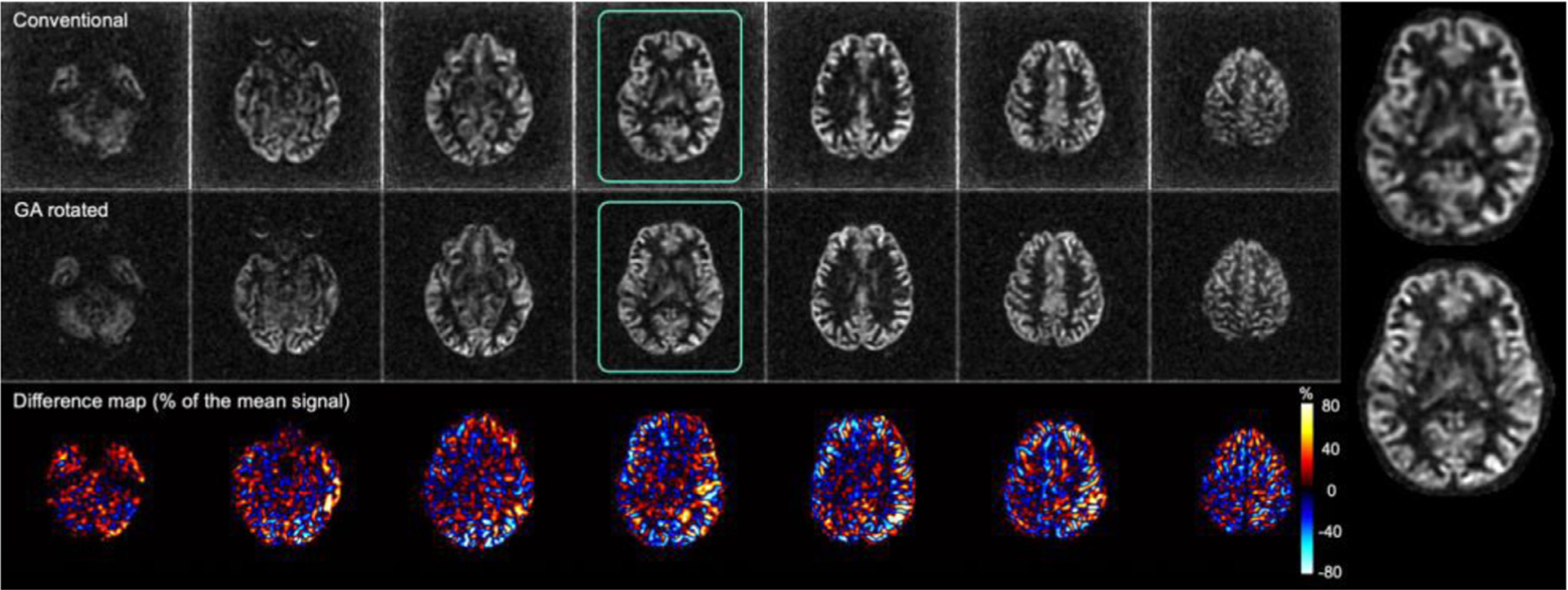
Comparison of full time-averaged perfusion-weighted subtraction images acquired with conventional interleaving and those from the GA rotated acquisition. Perfusion-weighted subtraction images were manually windowed to highlight cortical features and remove background noise.

**Fig. 6.**
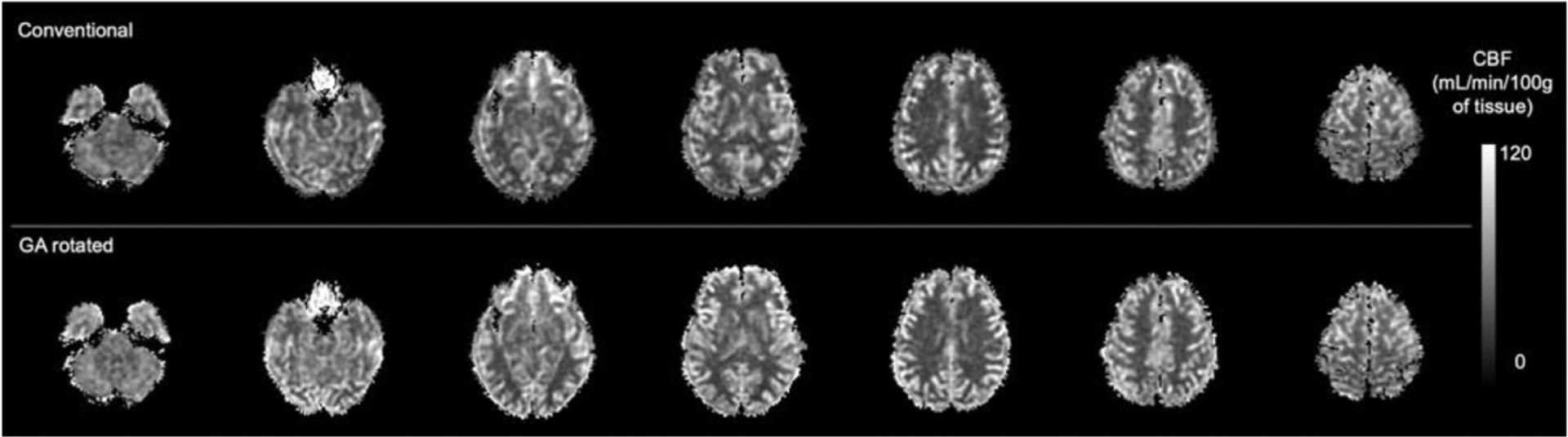
Comparison of CBF maps calculated for the conventional acquisition and the GA rotated acquisition. A quantitative grayscale map was used to facilitate quantitative comparison.

GA rotated acquisitions compared favorably with the conventional acquisition (Fig. 5). GA rotated acquisitions appear slightly higher resolution, consistent with the larger k-space radius reached by the variable density spirals. This is confirmed by the non-reference blurring metric which was significantly lower (*p* < 0.0001) for the GA rotated acquisition, 0.251 (SD = 0.013), than for the conventional acquisition, 0.270 (SD = 0.017). Direct subtraction of the two volumes shows they have very similar image intensities. Brain and subject averaged NRMSE between the images was 19.4% (SD = 4.2%). Corresponding CBF maps (Fig. 6) showed an average increase of 6.2 mL/min/100g of tissue across subjects for the CBF map calculated for the GA rotated acquisition (SD = 3.5 mL/min/100g of tissue, *p* < 0.0001).

### 3.2. Single-shot Imaging

**Fig. 7.**
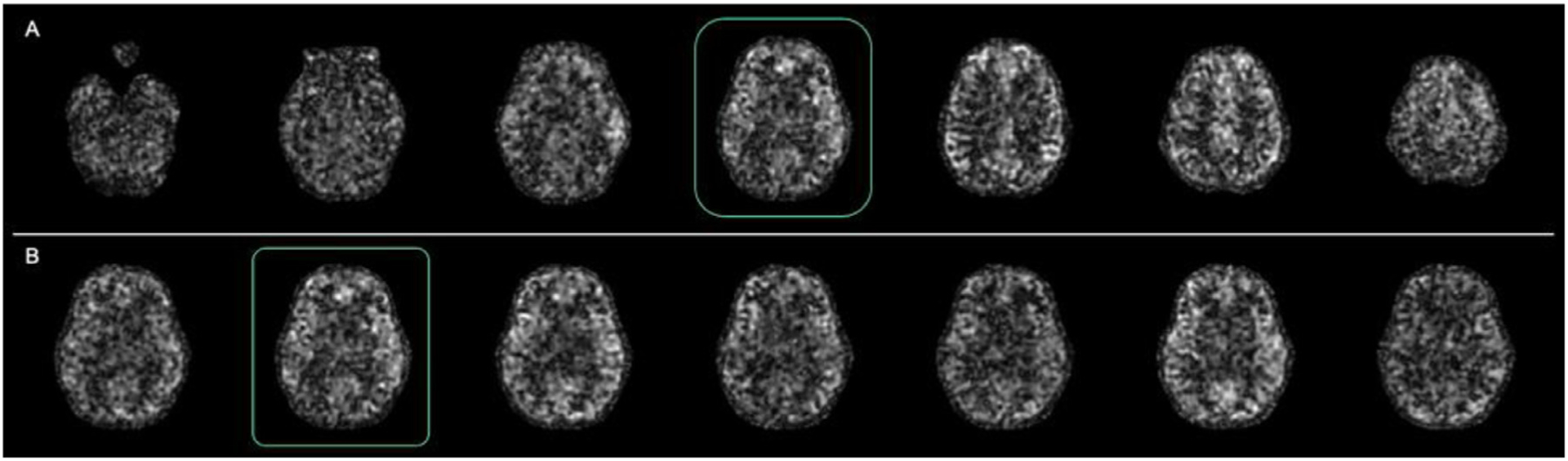
Single-shot ASL images reconstructed with CS - A) Different slices from the same time-resolved volume and B) Same slice over time.

**Fig. 8.**
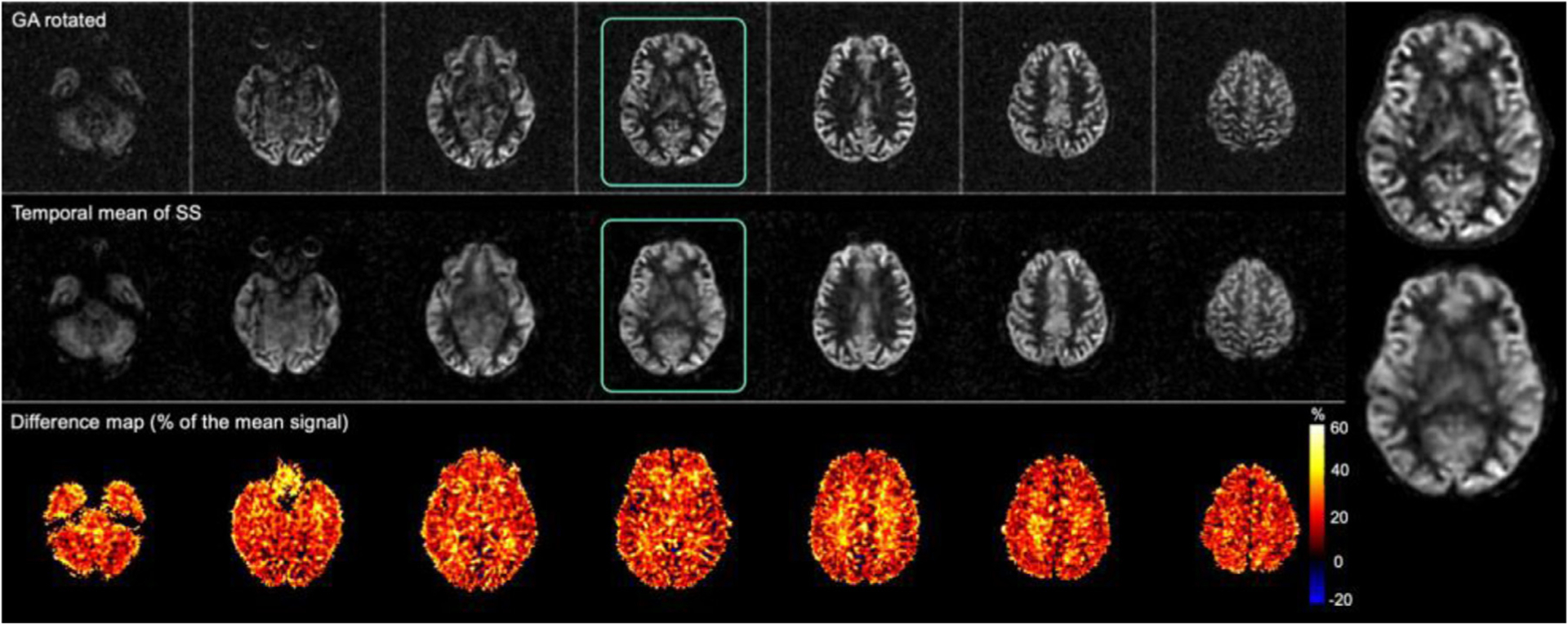
Comparison of full time-averaged GA rotated perfusion-weighted subtraction images and temporal mean of 39 single-shot images.

Reconstruction of a time series of 39 single-shot images required approximately 50 min on an iMac Pro (Apple Computer, Cupertino CA) with 6-core Intel Xeon W, 64Gb RAM and without GPU acceleration. CS reconstruction of ASL difference images from single-shot label and control pairs yielded recognizable brain images, though with much lower image quality than the time average reconstructions (Fig. 7). This image quality is consistent with the effects of reduced averaging on SNR and the appearance of physiologic fluctuations, such as those in resting-state networks, but also includes some level of degradation due to image undersampling and CS reconstruction. To better isolate these two contributions, we compared the magnitude average of the 39 CS reconstructions to the reconstruction of the fully sampled data set (Fig. 8). Time averaging greatly improves the appearance of the single-shot images but subtle blurring and other loss of features are still apparent in the CS reconstructions while the GA rotated images look sharper as seen in the enlarged slice. This is confirmed quantitatively by the blurring metric, which averaged 0.314 (SD = 0.019) for the time average CS images compared to 0.251 (SD = 0.013) for the fully sampled reconstruction (*p* < 0.0001). NRMSE difference between the two volumes was 11.8% (SD = 1.5%).

**Fig. 9.**
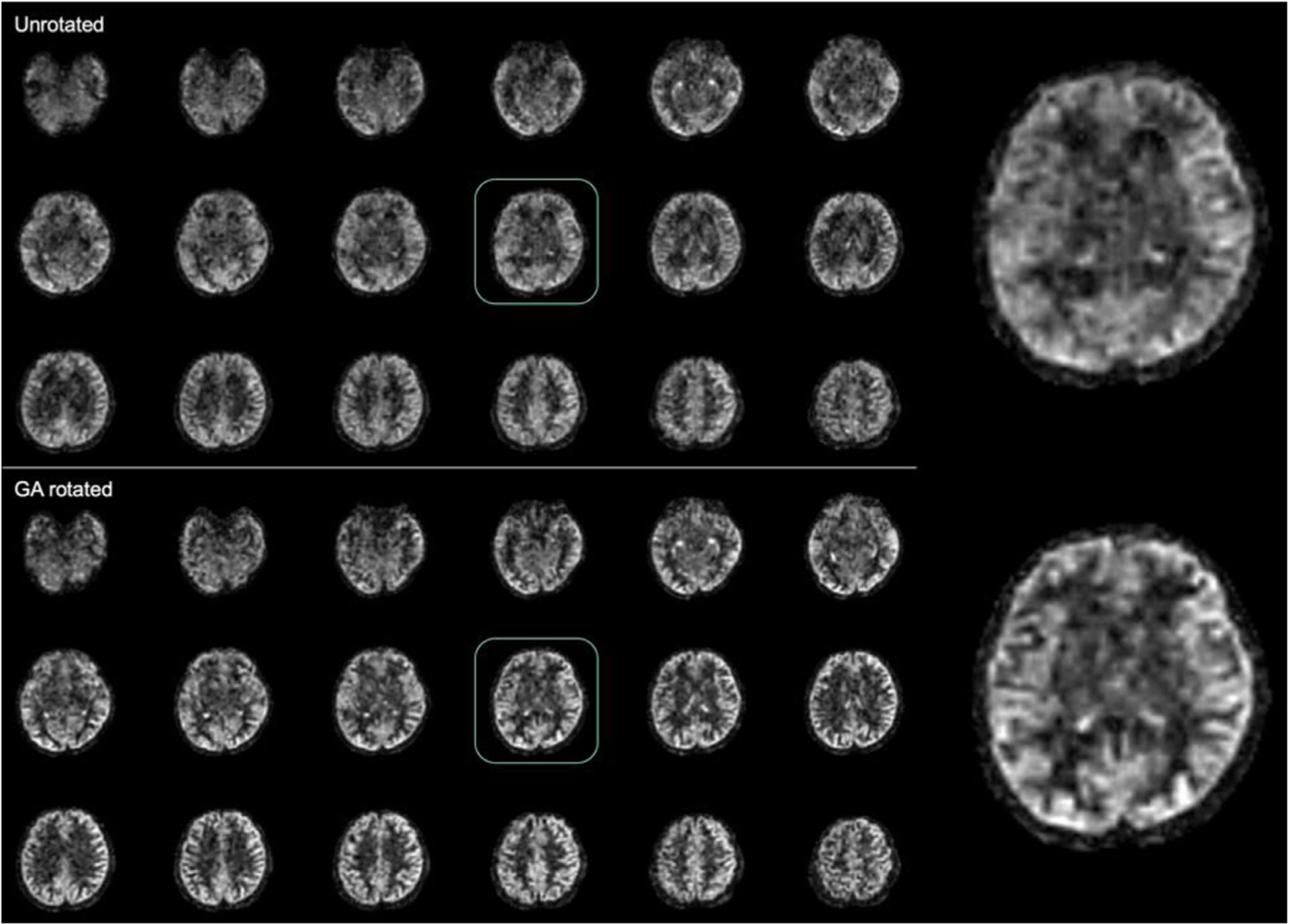
Comparison of three-shot average of simulated single-shot acquisitions with no rotation and approximate golden-angle rotation of the spiral waveform across slice encodes.

The image quality benefits of the GA rotated spiral trajectory for high undersampling acceleration were clearly apparent when comparing to the simulated unrotated data reconstruction (Fig. 9). The unrotated trajectory (without rotation across slice encodes) results in blurring, signal dropouts and coherent artifacts that are visibly apparent in the images. Such degradation is not observed in the GA rotated acquisition (Figs. 8 and 9), supporting the benefits of the latter.

### 3.3. Extraction of resting-state networks

**Fig. 10.**
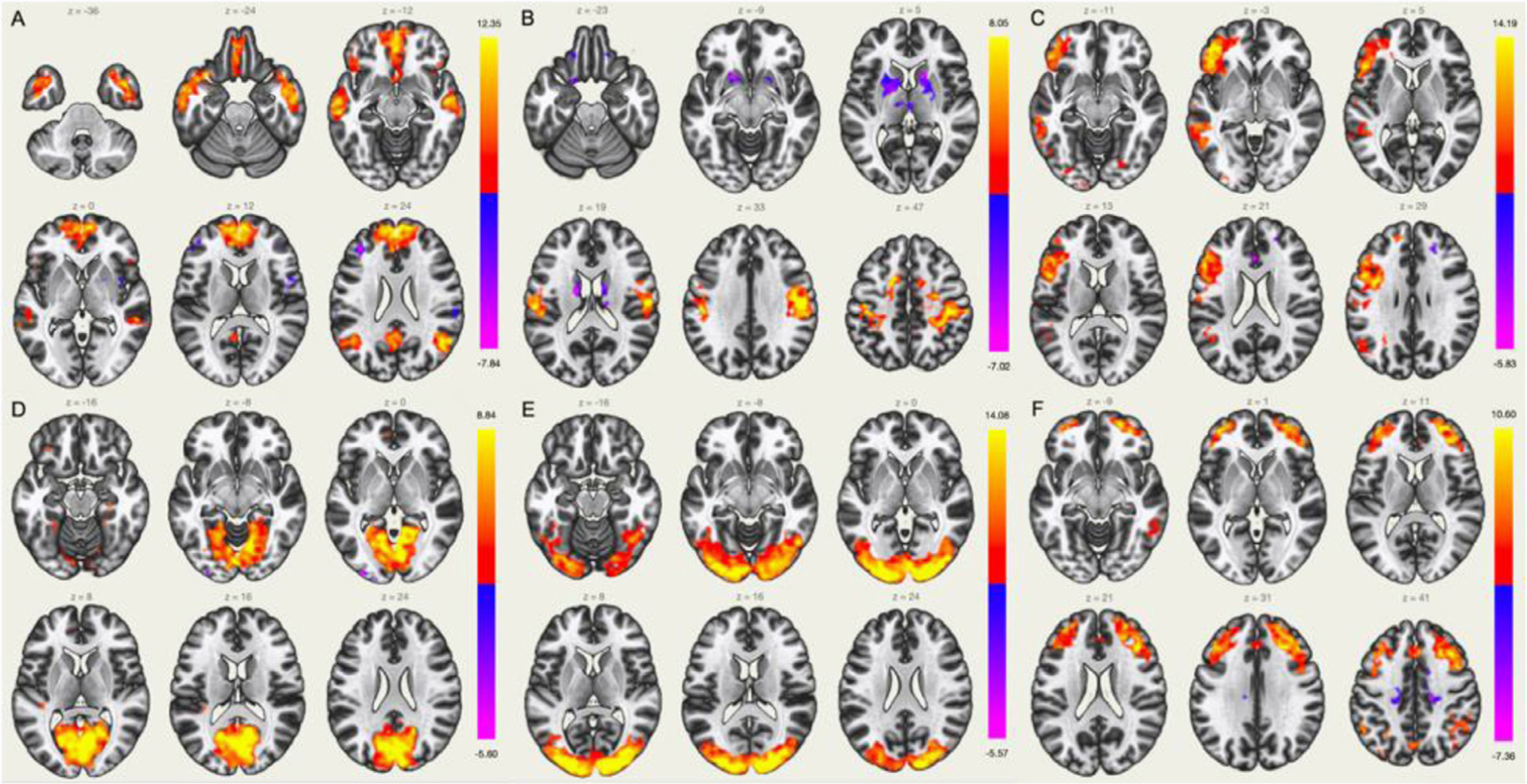
Independent components (A - DMN, B - sensori-motor, C - left lateral, D - medial visual, E – lateral visual, F – fronto-parietal) extracted from ICA analysis performed with the CONN toolbox.

Among the 11 ICs investigated using ICA analysis, well-known resting-state networks were extracted: default-mode network (DMN), medial visual, lateral visual, sensori-motor, left lateral, fronto-parietal control among others. Some of them are displayed in Fig. 10.

As previously described, we chose two specific ROIs to perform seed-to-voxel connectivity analyses.

**Fig. 11.**
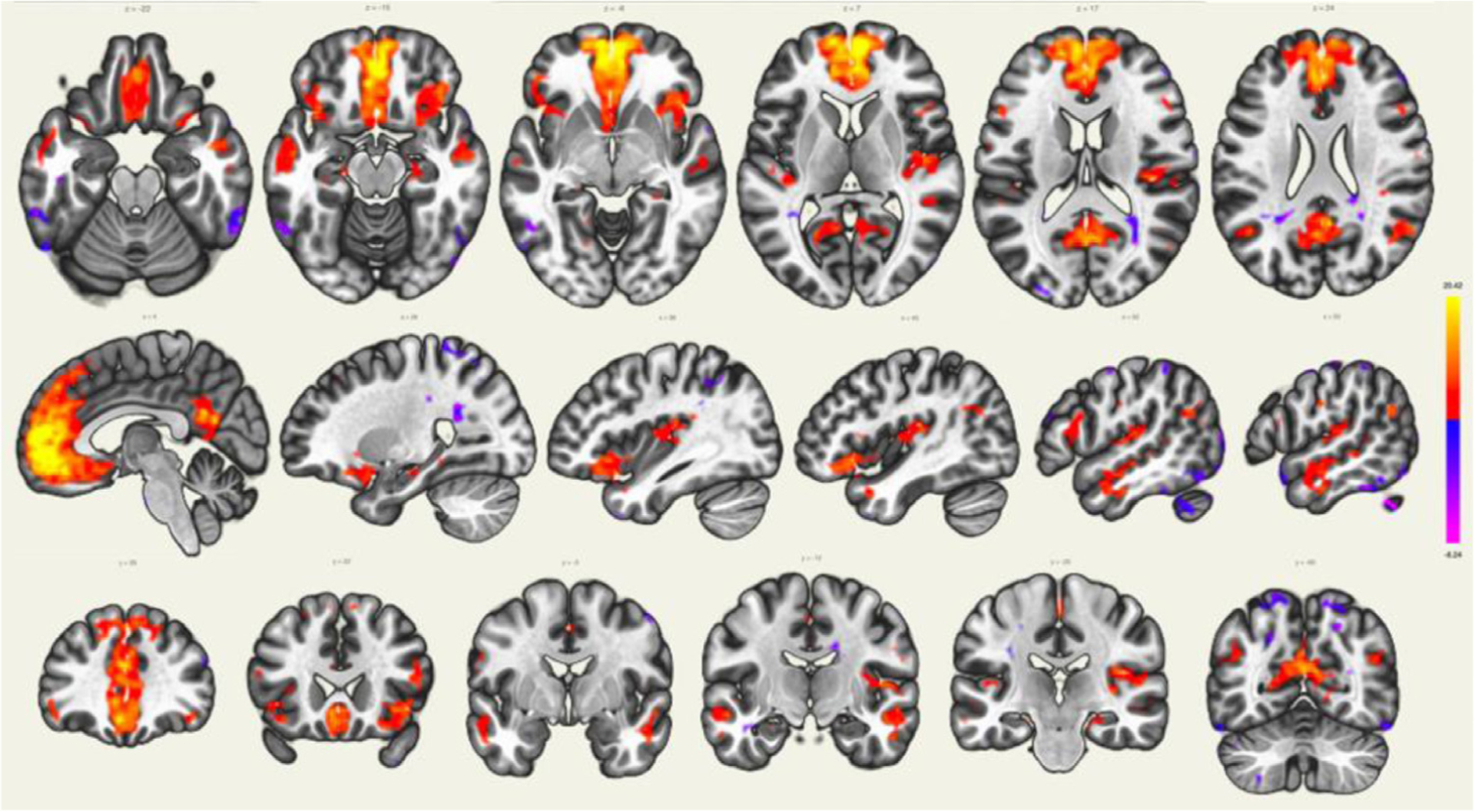
Seed-to-voxel brain connectivity analyses across subjects using the medial prefrontal cortex (part of the DMN) as a seed (red-yellow scale represents positive connectivity values and blue-purple scale represents negative connectivity values).

As displayed on Fig. 11, the MPFC is connected to the posterior cingulate cortex (PCC) and inferior parietal lobule (IPL), other regions involved in the DMN. The DMN also showed extensions in the orbitofrontal cortex and inferior temporal lobes.

**Fig. 12.**
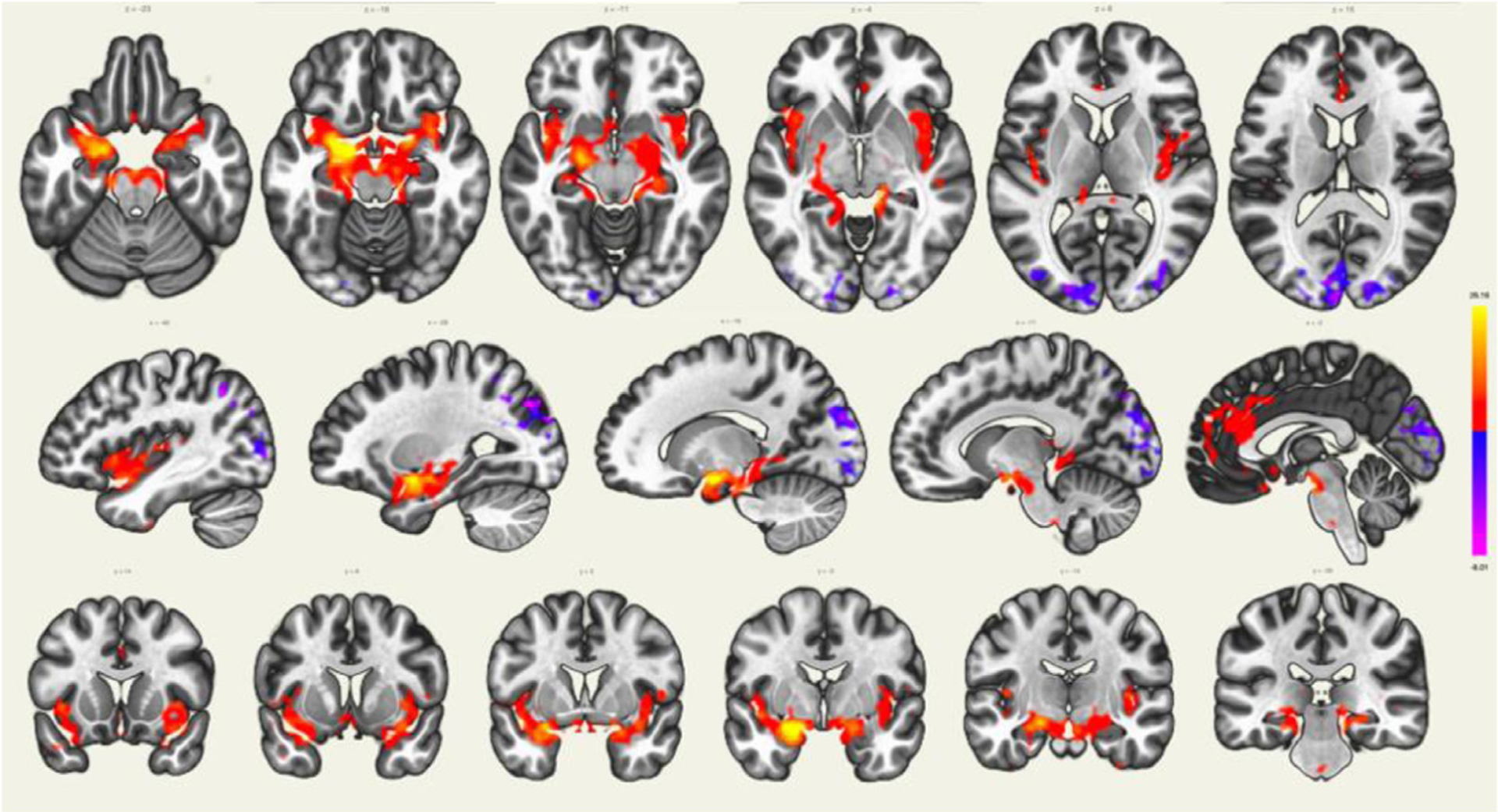
Seed-to-voxel brain connectivity analyses across subjects using the left amygdala as a seed (red-yellow scale represents positive connectivity values and blue-purple scale represents negative connectivity values).

The amygdala belongs to the affective network and is thus connected to the hippocampus, the insula and the anterior portion of the cingulate cortex. The amygdala is also connected to the subcallosal cortex which modulates its emotion regulation (Fig. 12).

## 4. Discussion

Our results emphasize that single-shot temporal resolution for dynamic ASL can be achieved without compromising the quality of traditional, multi-average multi-interleave time averaged blood flow acquisitions. This means such an approach can be introduced in any clinical and research acquisition. This would enable study of the blood flow modulations at rest, potentially both from vasoregulatory changes and from correlated neural activity. In some pathologies, fluctuations may be more diagnostic than mean blood flow values (Dai et al., 2020). Of course, modulations of blood flow may also be stimulated for diagnostic and research purposes, such as by CO_2_ (Last et al., 2007) or acetazolamide (Detre et al., 1999; Dai et al., 2020), blood pressure cuffs (Panerai et al., 2001), respiratory protocols (Detre et al., 1999; Puig et al., 2019), and electrical (Zheng et al., 2011) or task based (Kim, 1995) brain activity stimulations. Our methods should help to improve and support such studies.

The Stack of Spirals RARE employed in our study may have advantages over other 3D techniques for single-shot, undersampled acquisition with compressed sensing reconstruction. In particular, spiral acquisition allows for variable density acquisition such that the center of k-space can be fully acquired in each shot. This is advantageous because large-scale, low resolution fluctuations dominate the signal changes in ASL (Zhao et al., 2019) and potentially many physiologic noise sources. The full sampling at k-space center also provides information that can potentially be used for coil calibration and motion correction within each shot, which will be explored in future work. Spiral can be accelerated with conventional parallel imaging (Chang et al., 2017), but the inclusion of the irregular sampling introduced by pseudo golden-angle rotation makes reconstruction with CS type regularization more effective. 3D GRASE, which has been the primary 3D alternative to SOS, can also be accelerated, but the regular timing of the gradient echoes and the associated fixed off-resonance phase shifts complicates irregular sampling. Recent work (Spann et al., 2019) using CAPIRINHA accelerated 3D GRASE for ASL did not employ irregular sampling but employed temporal regularization to recover image quality. The effect of the regularization on sensitivity to dynamic changes was not evaluated. 3D CS sampling strategies for GRASE with modest, 3 gradient echoes, acceleration have recently been reported (Cristobal-Huerta et al., 2019) and may show the way for higher gradient acceleration factors.

Application of the single-shot ASL acquisition to resting-state ASL measures demonstrated dynamic sensitivity and intriguing sensitivity in regions where BOLD may be challenging (Dai et al., 2016). This suggests that ASL could play a role in studies of resting-state fluctuations. Whether serving as a complement to BOLD in regions with susceptibility or other artifacts, providing quantification of resting-state changes in quantitative physiologic units, or potentially serving as a primary measure of resting-state fluctuations, dynamic ASL acquired without additional cost of time during an otherwise conventional time averaged ASL blood flow acquisition shows great potential. Though current slow reconstruction time may not be ideal for widespread use, recent developments with deep neural network reconstructions (Mardani et al., 2019) suggest much faster reconstruction speed, and potentially quality, is possible.

Our work is not without limitations. First, we did not exhaustively optimize CS methods for reconstruction. Our regularization parameter was chosen empirically and only one form of regularization, wavelet L_1_ norm, was evaluated. We also did not explore adding regularization in the time dimensions. As shown previously (Spann et al., 2019), temporal regularization can greatly increase the image quality of dynamic single-shot acquisitions. In this initial report, we chose not to introduce assumptions about the amplitude or power spectrum of temporal variations. Depending upon the application, temporal regularization may be able to further improve the quality of our results. Additionally, vascular signals could contribute to the extracted resting-state networks (Franklin et al., 2020) displayed in this study, especially the amygdala network in Fig. 12. Longer PLD and especially vessel suppression could have been used to reduce this potential confound (Zhao et al., 2019). Optimization of 3D accelerated acquisitions for single-shot ASL was only partially addressed in this work. Simulations and acquisitions of previously reported and potential other strategies for 3D acquisition would be required for a full optimization and comparison. Even in our comparison with the GA rotation, we only simulated unrotated acquisitions by data reordering. A real such acquisition would have enabled more detailed testing for eddy current effects and the advantages of GA rotation for CS. Comparison with previous work is challenging because the objectives and spatial resolution were different and would have distracted from the focus of this work. The 3D accelerated work of Chang *et al* (Chang et al., 2017) focused on shortening of the echo train to reduce slice blurring. The work of Zhao et al. (2015) emphasized combination of multiple delay images with a hemodynamic model. Finally, our presented pseudo golden-angle single-shot acceleration strategy relies on acquiring as many spin echoes as the number of slices. For higher resolution imaging, this could be a limitation, though single-shot acquisition with significantly higher resolution may not be feasible due to signal-to-noise limitations.

## 5. Conclusion

The combination of pseudo golden-angle rotation and compressed sensing reconstruction enables single-shot 3D dynamic ASL acquisition without compromising time averaged ASL image quality. This capability makes possible characterization of resting blood flow fluctuations in any protocol where more conventional 3D ASL is employed, without affecting scan time or quality. Application of this dynamic 3D ASL to resting-state network measurement highlights how dynamic acquisition can extend the capabilities of ASL and potentially complement BOLD studies of brain networks.
